# The Ideal Graduate Study Institution

**Published:** 1909-07-10

**Authors:** 


### Editor's Letter-Box.

### "THE IDEAL GRADUATE STUDY INSTITUTION."

*To the Editor of* The Hospital.

In reference to our preliminary article under the above title, published in our issue of July 3, we have received from Professor R. Kutner, director of the "Kaiserin Friedrich-haus" for the Promotion of Graduate Study in Medicine, Berlin, a letter, dated June 29, 1909, from which we publish the following extracts:—

". . . The paper gives a complete idea of our organisation and very accurately represents the present position. Only one datum is wanting. Last year (March 1908) the committee of the several allied States (Bundesstaaten)—*i.e.*, the Prussian Committee (Central Committee), the Bavarian Committee, the Baden Committee, the Saxon Committee, and the Würtemberg Committee, as also the committees of the smaller allied States—have united to form an Imperial Commission for the Promotion of Graduate Study in Medicine (Fortbildungwesen). As you will see from the enclosed statutes of the Imperial Commission, the latter also has its headquarters in the Kaiserin-Friedrich-haus. The most important advantages thereby obtained are:—

"(1) That not merely the Ministries of Education of the individual allied States, but the Empire as such supports our endeavours with counsel and active help. In order to show his personal interest, His Majesty the German Emperor has lately contributed Mk. 10,000 towards our endeavours out of his 'Dispositionsfonds.'

"(2) That now the arrangements for the higher education of doctors in the whole German Empire can be made on a uniform plan. Possibly you can, on a suitable occasion, add the information that an Imperial Commission for the Promotion of Graduate Study in Medicine has been formed in our country.

". . . In a few days the last annual report on the Promotion of Graduate Study in Medicine in the German Empire will be ready (either the end of this or the beginning of next week); I shall then take the liberty of sending you a copy."

